# Deciphering the Shared and Specific Drug Resistance Mechanisms of Anaplastic Lymphoma Kinase via Binding Free Energy Computation

**DOI:** 10.34133/research.0170

**Published:** 2023-06-19

**Authors:** Yang Yu, Zhe Wang, Lingling Wang, Qinghua Wang, Rongfan Tang, Sutong Xiang, Qirui Deng, Tingjun Hou, Huiyong Sun

**Affiliations:** ^1^ Department of Medicinal Chemistry, China Pharmaceutical University, Nanjing 210009, Jiangsu, P. R. China.; ^2^ Innovation Institute for Artificial Intelligence in Medicine of Zhejiang University, College of Pharmaceutical Sciences, Zhejiang University, Hangzhou 310058, Zhejiang, P. R. China.

## Abstract

Anaplastic lymphoma kinase (ALK), a tyrosine receptor kinase, has been proven to be associated with the occurrence of numerous malignancies. Although there have been already at least 3 generations of ALK inhibitors approved by FDA or in clinical trials, the occurrence of various mutations seriously attenuates the effectiveness of the drugs. Unfortunately, most of the drug resistance mechanisms still remain obscure. Therefore, it is necessary to reveal the bottom reasons of the drug resistance mechanisms caused by the mutations. In this work, on the basis of verifying the accuracy of 2 main kinds of binding free energy calculation methodologies [end-point method of Molecular Mechanics with Poisson-Boltzmann/Generalized Born and Surface Area (MM/PB(GB)SA) and alchemical method of Thermodynamic Integration (TI)], we performed a systematic analysis on the ALK systems to explore the underlying shared and specific drug resistance mechanisms, covering the one-drug-multiple-mutation and multiple-drug-one-mutation cases. Through conventional molecular dynamics (cMD) simulation in conjunction with MM/PB(GB)SA and umbrella sampling (US) in conjunction with contact network analysis (CNA), the resistance mechanisms of the in-pocket, out-pocket, and multiple-site mutations were revealed. Especially for the out-pocket mutation, a possible transfer chain of the mutation effect was revealed, and the reason why different drugs exhibited various sensitivities to the same mutation was also uncovered. The proposed mechanisms may be prevalent in various drug resistance cases.

## Introduction

Anaplastic lymphoma kinase (ALK), as a member of tyrosine receptor kinase family, was first identified as a fusion partner in anaplastic large cell lymphoma (ALCL) in 1994 [1]. In the following decades, it was proven that ALK fusion proteins induced the constitutive activation of the ALK tyrosine kinase via the oligomerization of domains and led to numerous different malignancies [2–7]. By targeting ALK [8], the tyrosine kinase inhibitors (TKIs) are proved to be effective in the treatment of the non-small cell lung cancer (NSCLC). However, the targeted therapy often ends up with acquired resistance and clinical evasion of the drugs, where drug failure usually involves the occurrence of secondary mutations in the catalytic domain of ALK [9]. To date, a total of 3 generations of ALK-TKIs have been approved by Food and Drug Administration (FDA) in the United States. The usage of the first-generation ALK TKI, crizotinib, greatly improves the progression-free survival and total survival of patients with NSCLC, compared with the treatment of traditional chemotherapy [10,11]. Compared to crizotinib, the second-generation ALK TKIs represented by ceritinib exhibit better response rate for several drug resistance mutations such as L1196M and I1171N [12]. The third-generation ALK-TKI lorlatinib is able to overcome a majority of secondary mutations that lead to resistance to the first- or second-generation TKIs [12]. Unfortunately, further mutations may probably occur after the usage of lorlatinib for a period, causing drug resistance to the third-generation TKIs [12,13]. Therefore, at present, new generation of ALK inhibitors is still expected to be designed. Therefore, elucidating the shared and specific mechanisms behind the TKIs’ resistance is of great significance for anti-resistance drug design.

Mechanisms explaining drug resistance can roughly be divided into 2 categories, namely, the target-dependent and target-independent mechanisms [14]. Like the gatekeeper mutation T790M in epidermal growth factor receptor (EGFR) that impairs the binding between the drug and the protein directly [or enhances the binding between adenosine triphosphate (ATP) and the protein directly] [15], the L1196M mutation in ALK impairs a variety of inhibitors’ binding as well [9], which is a typical target-dependent mechanism. Moreover, the target-dependent mechanism may reflect in the overexpression of the ALK gene as well [16]. The target-independent resistances usually function through other biological mechanisms, including bypass signaling pathways [16–18], histological transformation [19,20], and drug efflux [21–23]. In the study of drug resistance, biological techniques can determine the progress of disease and the effect of drugs in the most direct ways; however, biological experiments are rather expensive, and there are still numbers of underlying resistance mechanisms that remained to be explored. Alternatively, due to the deepening understanding of structural biology and improvements of computer hardware and algorithms, the in silico computation methods make it possible to elaborate biological effects. Based on the publicly reported crystal structures and available activity data, computational predictions allow researchers to understand the dynamic structural alternations of drug–target interactions.

**Fig. 1.**
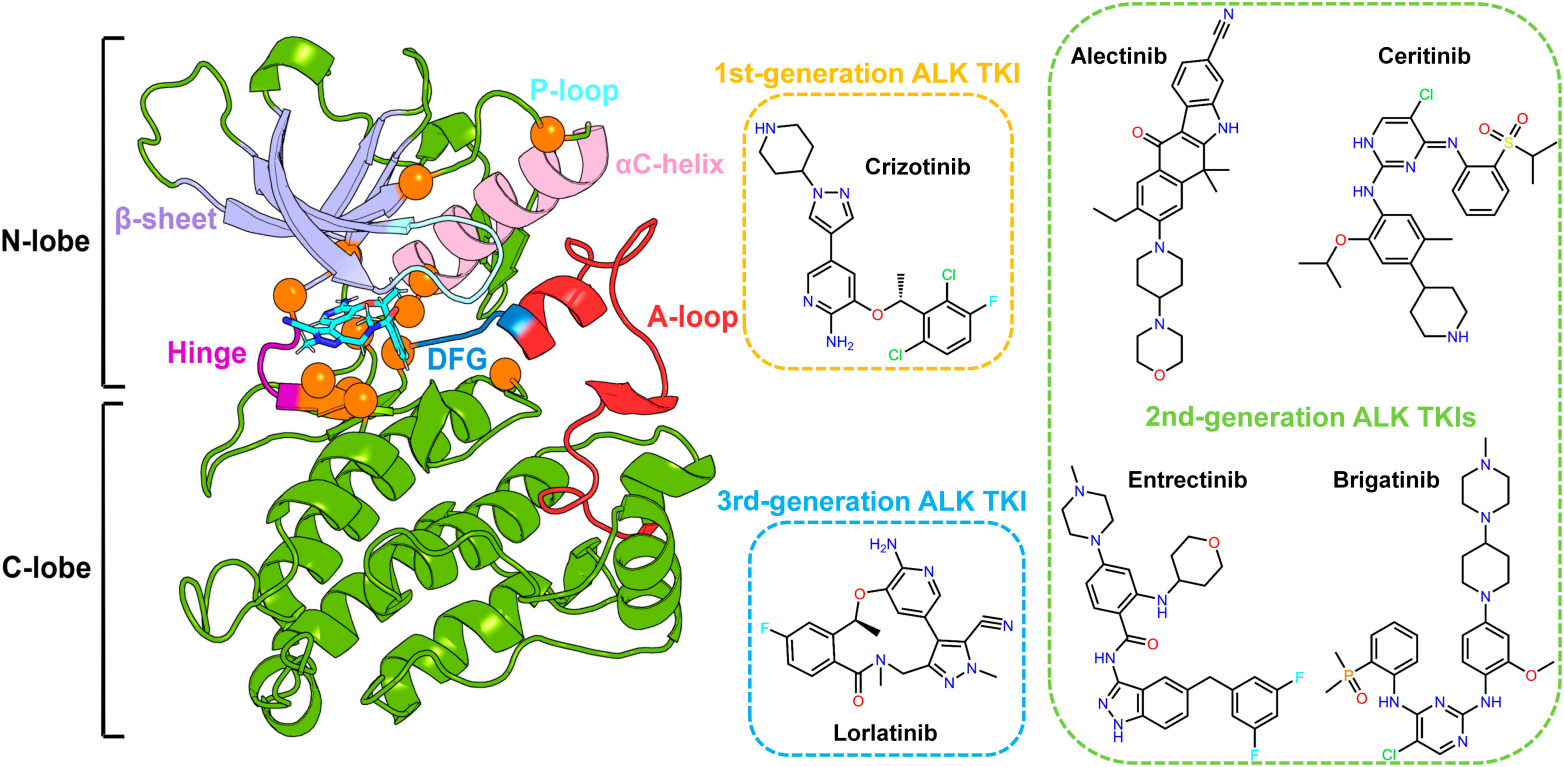
Structure of ALK protein and the 6 clinical drugs, where the mutated residues are presented in orange spheres.

Structurally, ALK holds a classical spatial structure shared by receptor tyrosine kinases and it can be roughly divided into 2 parts, namely, N-lobe and C-lobe (left panel of Fig. 1). The N-lobe mainly consists of 5 antiparallel β-sheet (β1 to β5; light purple region in Fig. 1) together with a regulatory αC-helix (pink region in Fig. 1). Between β1 and β2, there exists a small glycine-rich loop called P-loop (or G-loop; light blue region in Fig. 1), whose conformation influences the binding of ATP or ATP-competitive inhibitors. The C-lobe is dominated by 8 conserved α-helix and 4 short β-sheet motifs. The motif next to β6 (orange region in Fig. 1) is often referred to as the catalytic loop that stabilizes the magnesium ions when binding with an ATP. The binding site (ATP pocket) is located exactly at the cleft between the N-lobe and C-lobe (cyan region occupied by a TKI in Fig. 1). Nearby the binding site, there exists a conserved activation loop (A-loop; red region in Fig. 1) starting with the DFG motif (blue region in Fig. 1), which functions as a switch to regulate the activation or inactivation of a kinase. By targeting the ATP pocket of ALK with TKIs, a series of secondary mutations have been found occurring with resistance to the ALK drugs (orange spheres in Fig. 1).

Currently, a number of studies have clarified the ALK resistance mechanism through various approaches. For example, Ni et al. [24] have studied the mechanism of ceritinib resistance caused by F1174C mutation using conventional molecular dynamics (cMD) simulation with MM/GBSA binding free energy calculation, and found that the aromatic interaction network in the ALK-ceritinib system was destroyed by the mutation, which affected the conformational dynamics of the P-loop region, and finally weakened the binding of ceritinib to the protein. Chen et al. [25] have performed multiple replica Gaussian accelerated molecular dynamics (MR-GaMD) simulation with MM/GBSA to illustrate the drug resistance mechanism of mutations toward crizotinib. Through binding free energy analysis, they found that L1198F, L1198F/C1156Y, and C1156Y mutations significantly enhanced the flexibility of the protein and influenced the position of crizotinib along with the orientation of the protein, which significantly reduced the drug resistance. Besides, our group have also revealed the drug resistance mechanisms of a number of mutations to crizotinib, including C1156Y [26], L1152R, G1202R_ALK_/G2032R_ROS1_ [27], and S1206Y [28], by using enhanced sampling methods, finding that the conformational change of the P-loop region may be the major factor responsible for crizotinib resistance. Together, we have also developed a series of high-activity ALK inhibitors to combat the drug resistance [29,30]. Nevertheless, with the occurrence of various resistant mutations to all generations of drugs, it is urgent to fully uncover the underlying shared and specific drug resistance mechanisms. Therefore, in this study, on the basis of verifying the accuracy of computation methods [MM/PB(GB)SA and thermodynamic integration (TI)], we have investigated the resistance mechanisms of a series of representative mutations for the third-generation ALK TKI lorlatinib (the single-drug-multiple-mutation case; Table S1) and 6 launched or clinical trial drugs to one single mutation (the multiple-drug-single-mutation case; Table S2) to reveal the shared and specific drug resistance mechanisms. The results may help in further anti-resistant drug design.

## Results and Discussion

### Effects of multiple mutations on one single drug

Since a series of mutation data were collected for the third-generation ALK inhibitor lorlatinib, it is useful to investigate the performance of the various binding free energy calculation methods on the ALK-lorlatinib system. Therefore, in the following section, we first investigated the performance of end-point (MM/PBSA and MM/GBSA) and alchemical (TI) binding free energy calculation methods on the ALK-lorlatinib mutants, and then used the best protocol to analyze the impacts of several representative mutations to investigate the shared and specific drug resistance mechanisms.

### Performance of the end-point and alchemical binding free energy calculation approaches on the ALK-lorlatinib mutants

It is well known that the computational methods may involve system specificity, namely, different systems may show remarkable different results using the same predicting method [31–33]. To investigate the performance of the binding free energy calculation methods on the studied systems, MM/PB(GB)SA together with theoretically more rigorous method TI were employed to the systems consisting of lorlatinib binding to a variety of ALK mutants (including 28 single or double mutants; Table S1). Since a number of parameters may affect the predicting accuracy of MM/PB(GB)SA, here, we first investigated the most commonly used protocols and discussed the parameters that greatly impact the prediction accuracy, including the MD simulation time, dielectric constant, the selection of different polar solvation models (GB/PB), and whether to incorporate entropy to refine the results.

**Fig. 2.**
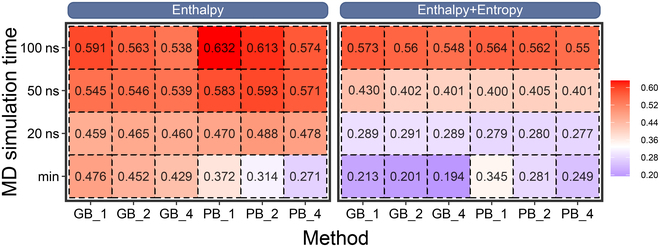
Impacts of MD simulation time, dielectric constant, and entropy on the performance of MM/PB(GB)SA. Pearson correlation coefficients are reported between the predicted binding free energy and the experimental data. The label “min” denotes the result based on the minimized structures.

As shown in Fig. 2, generally, the MD simulation time exhibits the most remarkable influence on the predicting accuracy, with the prediction accuracy proportional to the MD simulation time under any other conditions (dielectric constant or polar solvation model). The application of different polar solvation models (GB/PB) brings subtle difference in the result, where the GB model performs better only when the minimized structures were used, whereas with the increase of the MD simulation time, the PB model shows a better accuracy (with the best *r*_p_ = 0.632 at ε_in_ = 1 and 100 ns). Moreover, a lower dielectric constant (ε_in_ = 1) seems to work better in the ALK system, although no large difference is shown between different dielectric constants (*r*_p_ = 0.538 to 0.591 and 0.574 to 0.632, respectively, for MM/GBSA and MM/PBSA at 100-ns MD simulation time and ε_in_ = 1 to 4). Additionally, the incorporation of entropy effect into the binding free energy calculation may drive the predicted value closer to the experimental one. Here, we incorporated the normal mode entropy (NME) into the MM/PB(GB)SA results. Unfortunately, as shown in the right part of Fig. 2, the incorporation of NME to MM/PB(GB)SA impairs the prediction result in all cases, suggesting that it is better to analyze the mutation effects based on the enthalpy part of the system (∆*H* or effective binding free energy). Therefore, we analyzed the underlying drug resistance mechanisms with the enthalpy part of the end-point binding free energy in the following parts of this work.

**Fig. 3.**
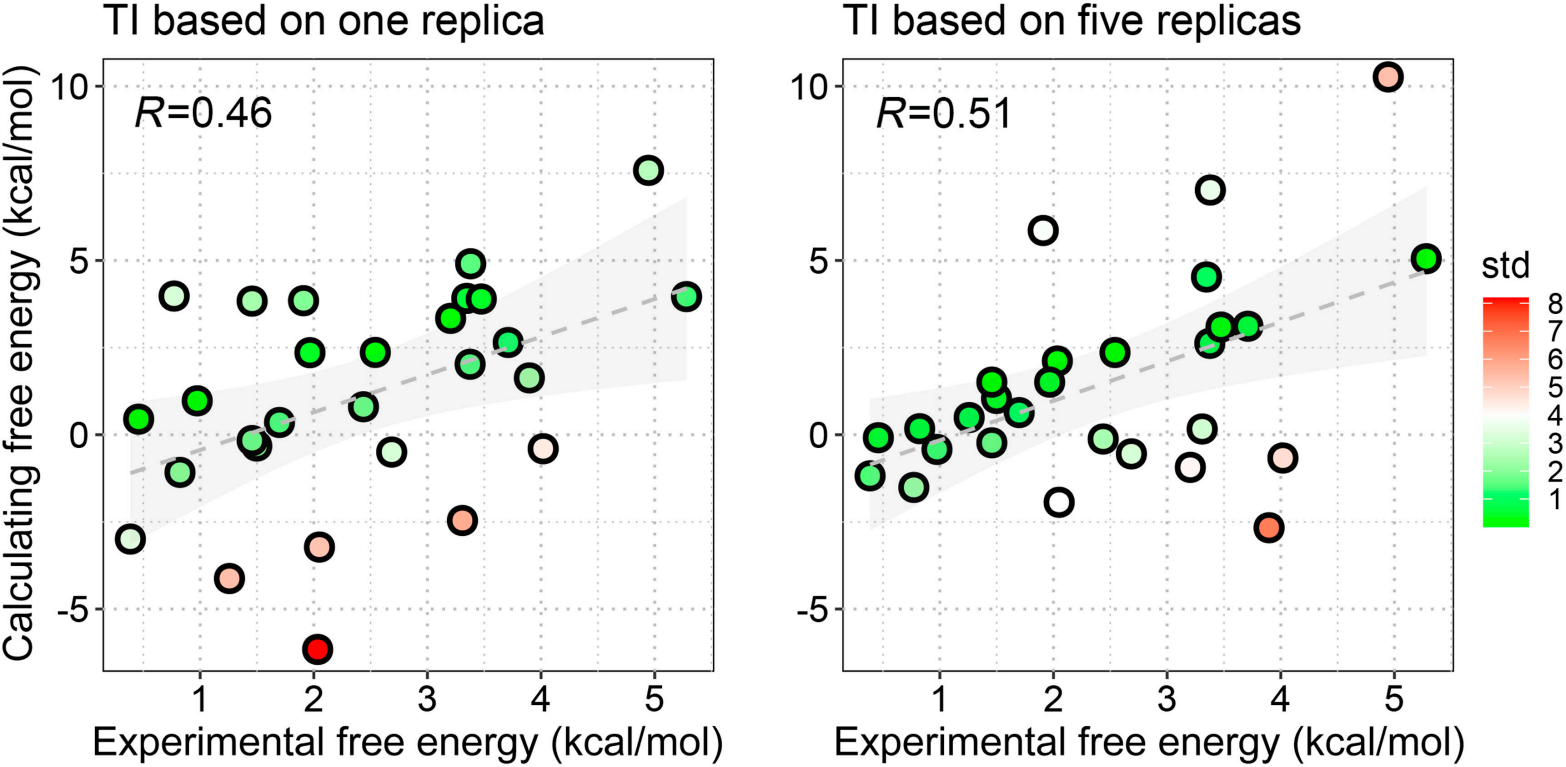
Accuracy of TI based on single- (left) and multiple-replica (right) protocols.

Furthermore, the theoretically more rigorous method TI was used to verify the accuracy of the computational method. Previous works suggest to use multiple short trajectories instead of a long trajectory [34], so in this work we adopted 2 strategies to perform TI, including (a) TI based on one single trajectory (one replica, 14 windows × 1 ns) and (b) multiple replicas (5 replicas, 14 windows × 1 ns × 5 rounds). As shown in Fig. 3, compared with the single replica strategy, the multiple-replica TI protocol improves both the Pearson correlation (*r*_p_ = 0.51 versus 0.45 for TI_5-replica_ and TI_1-replica_, respectively) and robustness of the resulted binding free energies, where a smaller absolute error [mean unsigned error (MUE) = |∆∆*G*_pred_ − ∆∆*G*_exp_|] is shown of the 5-replica TI strategy (MUE = 2.21 versus 2.65 kcal/mol for TI_5-replica_ and TI_1-replica_, respectively; Table 1).

**Table 1.** Mean unsigned error (MUE) of the 4 binding free energy calculation strategies.

Method	MUE (kcal/mol)
TI_1-replica_	2.65
TI_5-replica_	2.21
MM/GBSA_100ns&ε=1_	2.55
MM/PBSA_100ns&ε=1_	2.24

Compared with the alchemical method (TI_5-replica_), it shows that MM/PBSA can give a comparable absolute error (MUE = 2.24 versus 2.21 kcal/mol for MM/PBSA_100ns&ε=1_ and TI_5-replica_, respectively) and a higher Pearson correlation (*r*_p_ = 0.63 versus 0.51 for MM/PBSA_100ns&ε=1_ and TI_5-replica_, respectively) on the investigated systems. Moreover, absolute binding free energies were calculated by the end-point methods, which may help in analyzing the binding mechanism difference on individual mutants. Furthermore, a 5-replica TI calculation needs nearly twice as much simulation time as a 100-ns cMD-based end-point binding free energy calculation. Therefore, considering all, we decided to use the end-point method (MM/PBSA) for the drug resistance mechanism analyses.

### Drug resistance mechanism analyses of the in-pocket, out-pocket, and double mutations on the ALK-lorlatinib systems

With the best computational strategy (MM/PBSA) in hand, here we investigated the shared and specific drug resistance mechanisms for a series of representative mutants coving various drug resistance cases, including the in-pocket-, out-pocket-, and double-mutation cases.

#### Impact of the in-pocket mutation (L1198F)

**Fig. 4.**
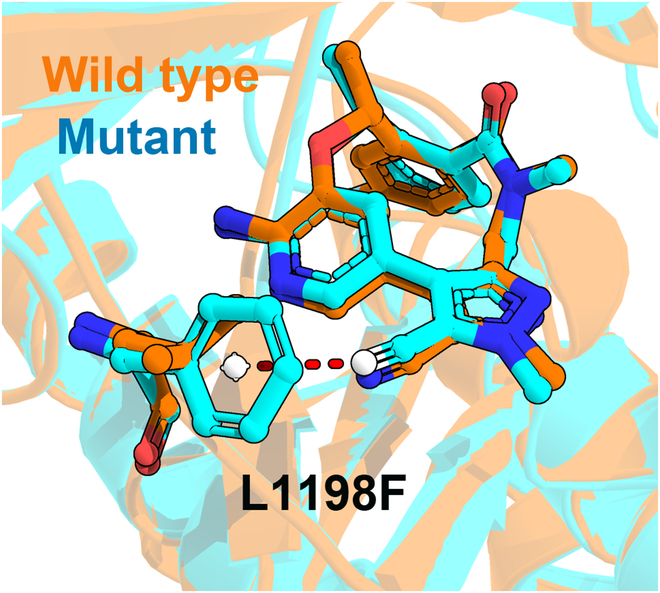
Binding mode of lorlatinib and residue 1198. The red dotted line represents the electrostatic repulsion effect.

Drug resistance generally occurs accompanied with single or multiple mutations, and these mutations can be classified based on their positions, such as in-pocket and out-pocket mutations. Generally, mutations occurring near the drug will always affect its binding directly. For example, in the kinase system, the interaction patterns between gatekeeper mutations and the TKIs usually limit the specificity of the inhibitors [35]. Similar to the gatekeeper mutations, other resistant mutations occurring near the binding site can hinder drugs’ binding directly as well. Take L1198F mutant as an example (where, compared with the wild-type ALK, the binding free energy of lorlatinib was attenuated by 1.45 and 2.79 kcal/mol in the experiment and our predicted result, respectively, in the L1198F mutant), the Cα atom of residue 1198 is positively charged because of the electron-withdrawing group, and after the aliphatic chain (L1198) is replaced by a benzene ring (F1198), the benzene ring of F1198 becomes negatively charged as well because of the conjugation effect, which is consistent with the atomic charges distributed on phenylalanine in the AMBER force field. As shown in Fig. 4, the -C≡N group of lorlatinib is perpendicular to the benzene ring, where the nitrogen atom is also negatively charged. Accordingly, the repulsive effect between -C≡N and the benzene ring hinders the drug–target interaction directly and thereafter leads to drug resistance, which is also consistent with the energy decomposition result that the electrostatic contributions (∆*E*_ele_) of L1198 and F1198 are −3.83 and −2.40 kcal/mol (Table S3), respectively, in the wild-type and mutated ALK. The resistance mechanism of in-pocket mutations may be the most understandable ones that usually share a similar underlying mechanism by directly hindering drugs’ binding.

#### Impact of the out-pocket mutation (I1171S)

Different from the in-pocket mutations, a larger number of drug resistance mutations are found occurring outside of the protein binding pocket, which may impair drugs' binding through indirect ways. In our dataset, there is one of the serious lorlatinib-resistant mutations, I1171S, whose resistant mechanism is of interest since the mutation site is located far away from the ligand (drug-mutation distance >8 Å, namely, outside the pocket), and may help us understand the shared resistant mechanism arising from the out-pocket mutations. In fact, results of 3 computation repeats showed that the binding free energy of lorlatinib in I1171S mutant was robust and attenuated by 1.86, 0.85, and 1.81 kcal/mol compared with that in the wild-type ALK (Table S4). Therefore, the case of I1171S can be served as a representative example for further analysis.

**Fig. 5.**
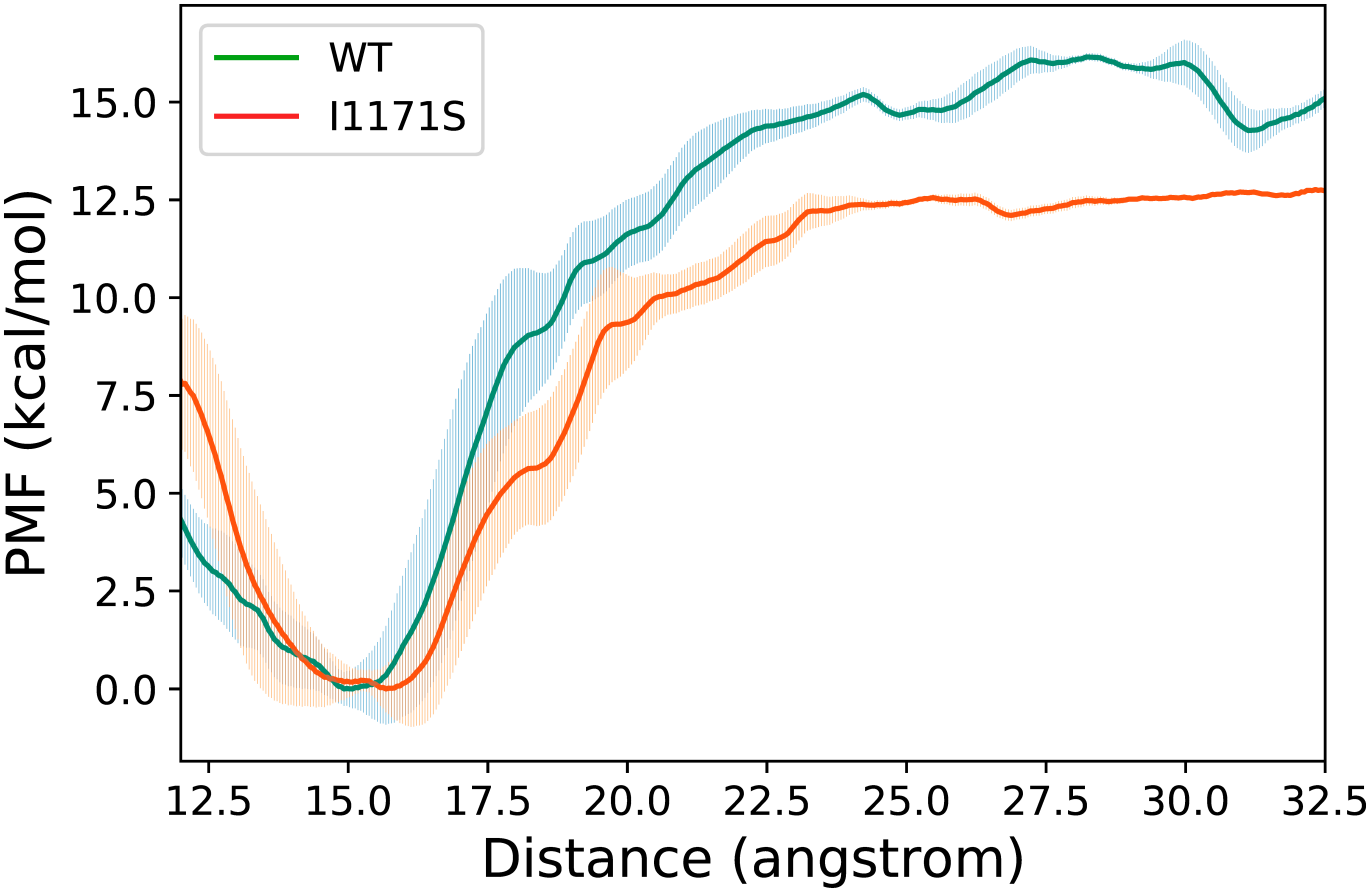
Comparison of the average PMFs of lorlatinib dissociating from the wild-type (green line) and I1171S (orange line) mutated ALK.

Generally, we infer that the effect of mutation is transmitted through the interaction between the surrounding residues, thus affecting the drug’s binding. However, it is usually difficult to capture the impact of a long-distance mutation in a short-time cMD simulation. The enhanced sampling method is able to promote the sampling in important degrees of freedom, thus capturing more clues to help find resistance mechanism. Therefore, here we investigated the resistance mechanism of the long-distance mutation (I1171S) through umbrella sampling (US). As shown in Fig. S1, after 18 rounds of US simulation, the potentials of mean force (PMFs) reach convergence in both the wild-type and the I1171S mutated systems. Thus, we derived the averaged PMF curves from the last 10 rounds of US samples. As shown in Fig. 5, the binding free energy of lorlatinib in the I1171S mutant decreases for 2.86 kcal/mol compared with that in the wild-type ALK, which is consistent with the wet-laboratory experiment (∆∆*G*_exp_ = 2.05 kcal/mol and our end-point computation; Table S4). Nevertheless, it is hard to analyze the binding/dissociating details of the drug with the PMF curves alone. Therefore, we analyzed the kinetic characteristics of the 2 systems based on the US trajectories.

**Fig. 6.**
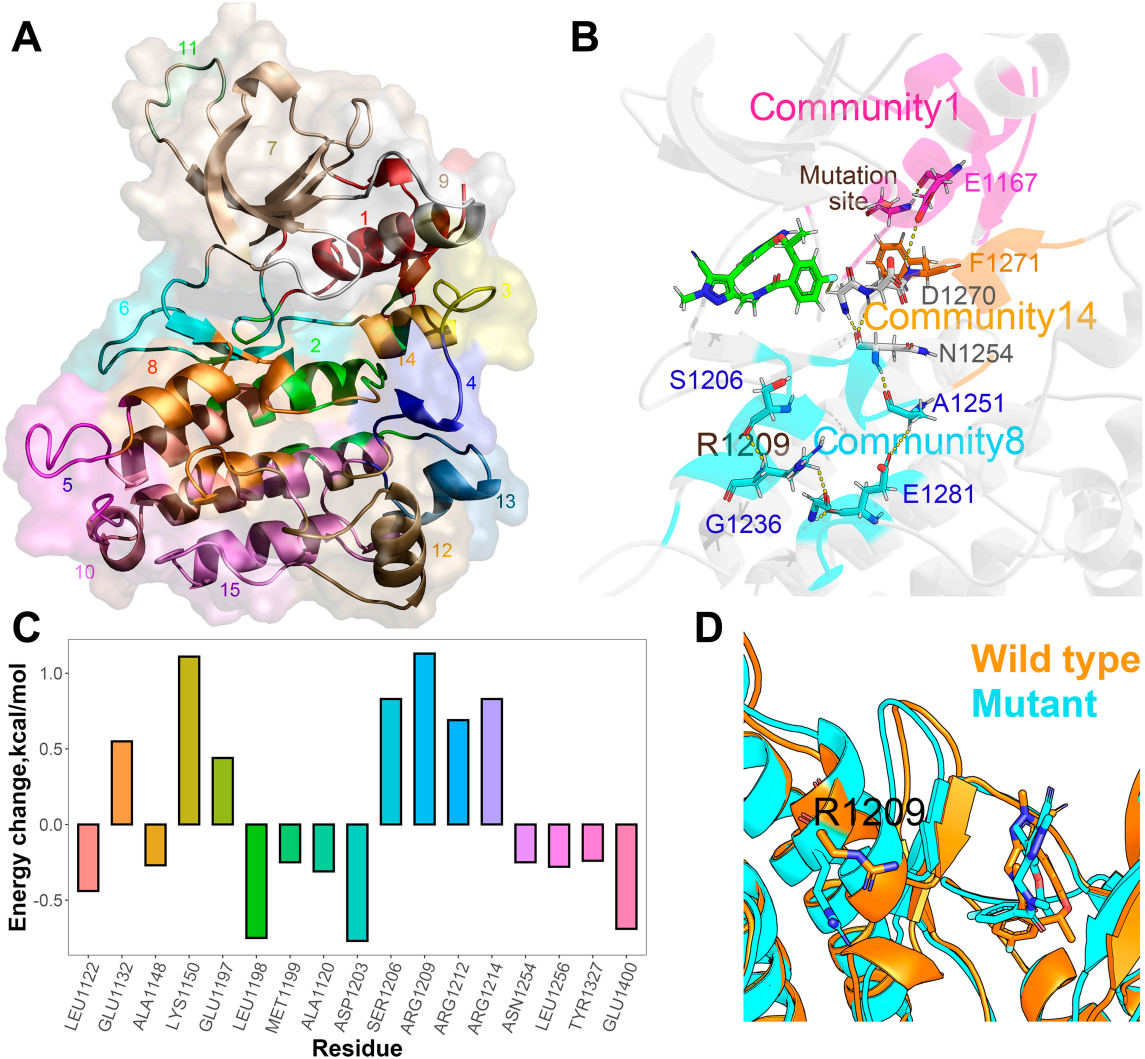
(A) Community distribution of the I1171S mutant. (B) Remote interaction chain among community 1/8/14. (C) Energetic change of residues in the wild-type and I1171S mutated systems (∆∆*G* = ∆*G*_MT_ − ∆*G*_WT_). (D) Comparison of the ligand binding modes in the wild-type (orange) and I1171S mutated (cyan) ALKs.

Based on the trajectories of US simulations, the contact network analysis (CNA) was carried out to find the motion cooperativity between different parts of the protein. As shown in Fig. 6A, except for the ligand, the mutated system (protein) could be divided into 15 communities, whereas there were only 9 communities in the wild-type system (Fig. S2), exhibiting the impact of mutation effect on the dynamic characteristics of the mutated system.

MM/PBSA decomposition was also conducted on the bound-state structures to reveal the clues causing the weakened binding capacity of the drug in the mutated system. It could be found that a region (residue 1206 to 1214) of community 8 (cyan in Fig. 6B) shows significant change in energetic contribution to the ligand in the ALK mutant (with each residue attenuated by 0.5 to 1 kcal/mol in the mutant; Fig. 6C). The results show that community 1 containing the mutation site (I1171S) is indirectly related to community 8 through community 14 (Fig. 6B). Therefore, an interaction chain (S1171-E1167-F1271-D1270-N1254-A1251-E1281-G1326-R1209-S1206) transferring mainly through H-bonds and salt bridges was dug out (Fig. 6B). The start of this transmission chain is the H-bond formed by the -OH group on the mutation site (S1171), which cannot be formed in the hydrophobic residue I1171 of the wild-type ALK (Fig. S3). This difference finally affects the region where community 8 is located through continuous transmission. Take R1209 as an example, which shows the largest energetic change upon the mutation (∆∆*G*_MT-WT_ = 1.14 kcal/mol; Table S5), the orientation of side chain of the residue is dramatically changed (cyan stick model, Fig. 6D) in the ligand binding/dissociating process compared with that in the bound state (orange stick model, Fig. 6D). Moreover, the orientation of the ligand (Fig. 6D) is influenced by the mutation in a similar transfer chain but much shorter, with the effect transferred to the ligand after N1254 through G1269 (S1171-E1167-F1271-D1270-N1254-G1269-lorlatinib).

In this section, we have elucidated the drug resistance mechanism of a representative out-pocket mutation (I1171S), which shows that a long transmission chain may be responsible for the effect of a long-distance mutation. This phenomenon has also been found in the case of the ALK C1156Y mutant, where the mutation is located >10 Å away from the binding drug (crizotinib), but also leads to serious drug resistance [26]. Therefore, although complicated and indirect, the revealed mechanism may be prevalent in protein-ligand interactions influenced by the out-pocket mutations because a common foundation (protein environment with crowded interactions) is shared by the systems.

#### Impact of double mutations (G1269A&I1171S)

The secondary drug resistance mutations often occur cumulatively with the ongoing treatment. For example, although both I1171S and G1269A single mutants of ALK cause moderate resistance to lorlatinib (with the attenuation of the binding affinity for 1.5 to 2.0 kcal/mol in the mutants; Table 2), the double-mutation mutant (I1171S&G1269A) impairs the drug’s binding more seriously than the 2 individual ones (attenuating the binding affinity of ~4 kcal/mol in experimental test; Table 2). Unfortunately, the resistance mechanism is still ambiguous. Consequently, energy decomposition was conducted to analyze the double-mutation system.

**Table 2.** Binding free energy difference and energetic contribution of E1197 in the single- and double-mutation systems (kcal/mol).

Mutation	∆∆*G*_exp_	∆∆*G*_pred_	Energetic contribution of E1197
G1269A	1.50	1.17	−1.41
I1171S	2.04	2.19	−1.67
G1269A&I1171S	3.98	14.44	1.29

**Fig. 7.**
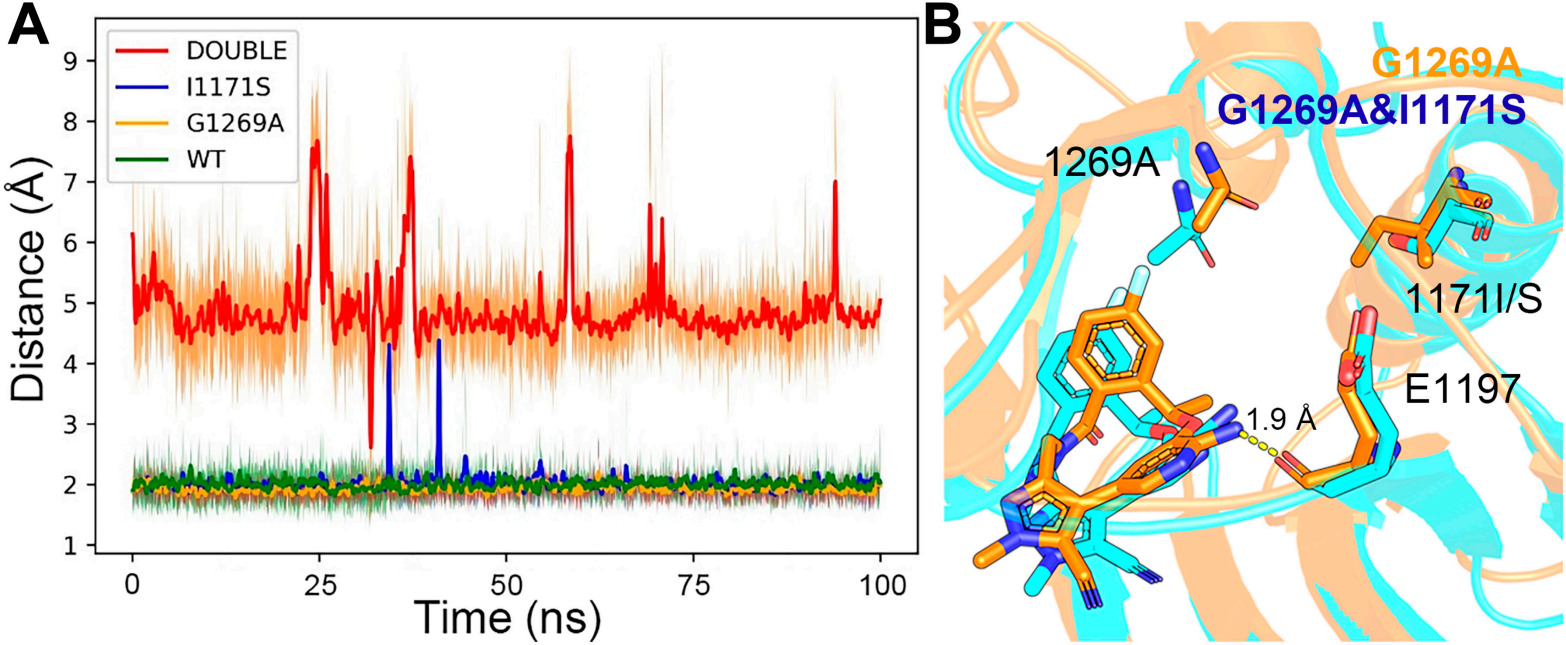
(A) Evolution of H-bond (hydrogen on the amino of lorlatinib and backbone oxygen on E1197) during the 100-ns cMD simulation in the wild-type (green) and mutated systems (blue, orange, and red for the I1171S, G1269A, and I1171S&G1269A mutants, respectively). (B) Average binding mode of lorlatinib in the G1269A (orange) and G1269A&I1171S (cyan) mutants of ALK, where the H-bond is colored in yellow dashed line.

As shown in Fig. S4, the energy decomposition spectrum shows that E1197 shows the largest energetic loss (~3 kcal/mol) to lorlatinib after double mutating, where residue E1197 shows favorable energetic contribution to the drug in the 2 single-mutation mutants (enhanced by ~−1.5 kcal/mol to I1171S and G1269A; Table 2), but the energetic contribution becomes unfavorable for binding of the ligand (~1.3 kcal/mol) in the double-mutation mutant (I1171S&G1269A). Further investigation shows that E1197 can form H-bond with lorlatinib in both the wild-type and single-mutation systems (green, blue, and orange lines, respectively, in Fig. 7A); however, the H-bond disappears in the double-mutation system (with the distance of the 2 H-bond-forming atoms >5 Å during most of the simulation time; red line in Fig. 7A). As shown in the averaged structures of the 2 systems, compared with the G1269A single-mutation mutant (orange in Fig. 7B) or the I1171S/wild-type systems (orange in Fig. S5), the I1171S&G1269A double mutations shows obvious “squeezing” effect to the ligand (cyan in Fig. 7B), forcing the ligand away from E1197, which hinders the formation of H-bond between them.

Taken all, compared with the single-mutation resistance, the double-mutation (or multi-mutation) mutant may further impair the drug’s binding capability with an aggregated (or cooperative) effect: On the one hand, it may hinder the drug–target interaction directly (such as attenuating the H-bond), and on the other hand, it may influence the kinetic property of the protein structure (such as influencing the signaling transmission chain in the I1171S mutant).

### Effects of one single mutation on multiple drugs

In the previous section, we have validated computational methods based on the lorlatinib-ALK mutants and analyzed drug resistance mechanisms of several distinct cases. But apart from lorlatinib, it is not clear whether other drugs have the same mechanism in binding with the ALK mutants. In this section, we investigated the effect of one single mutation on multiple drugs. Six ALK TKIs, including the first-generation inhibitor crizotinib, the second-generation inhibitors alectinib, ceritinib, brigatinib, and entrectinib, and the third-generation TKI lorlatinib, were collected for the analysis. The result may provide us a full picture of mutation effects on various cases of drug resistance.

#### Impact of the L1256F mutation on 6 clinical drugs

Cases have been found during treatment that the ALK mutation L1256F can lead to various resistance to the ALK drugs (with strong drug resistance to most ALK drugs; Table S2) [36]. Therefore, it is of interest to investigate the resistance or anti-resistance mechanism of L1256F to different ALK drugs. Similar to the research process above, we first investigated 4 computational strategies [including MM/PB(GB)SA under 100-ns cMD simulation and ε_in_ = 1 and TI with one or 5 replica(s) for the binding free energy calculation] in predicting the cases of one mutation effect to multiple drugs to derive the best protocol for the following drug resistance mechanism analysis.

**Fig. 8.**
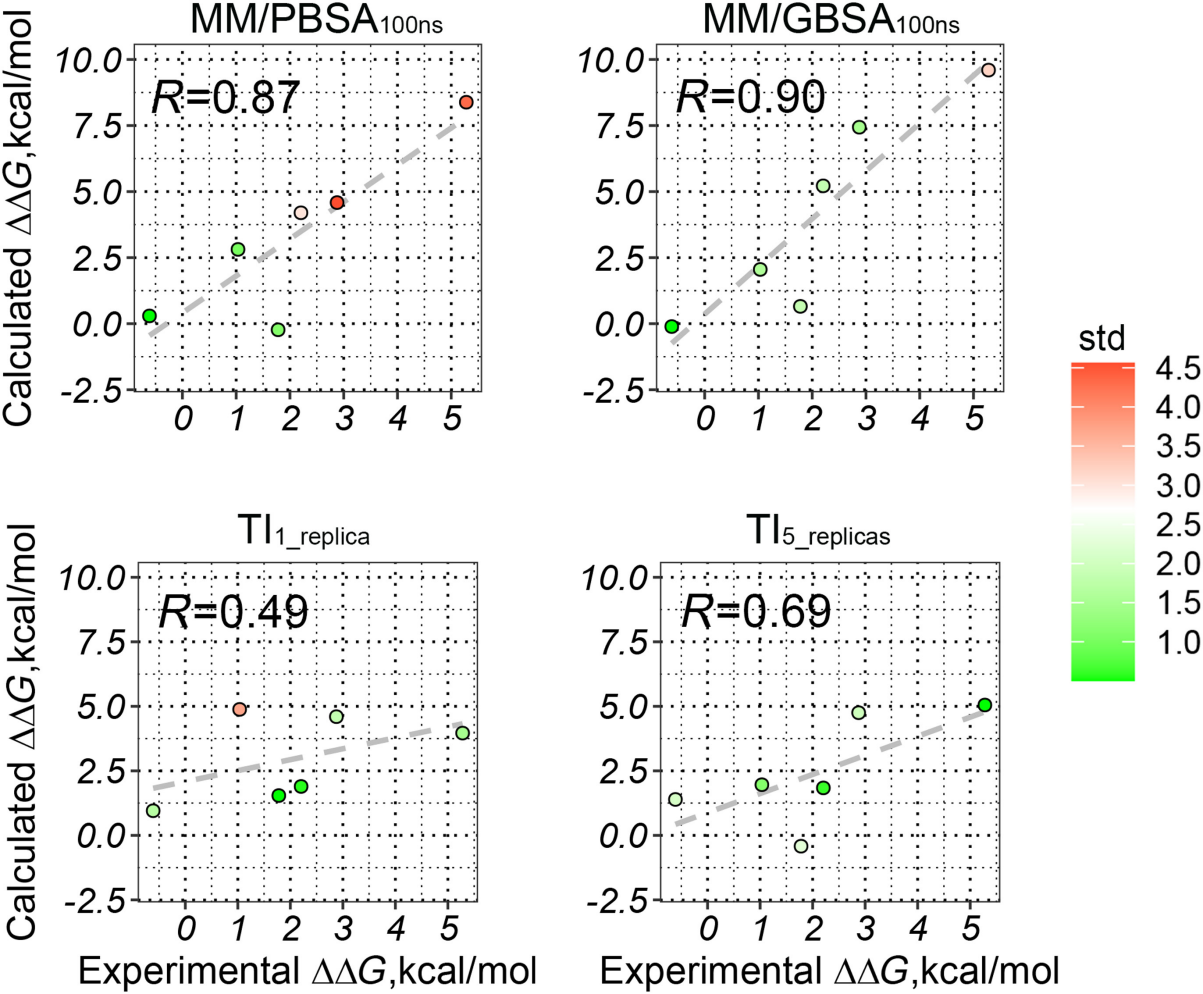
Pearson correlation of the predicted and experimental binding affinity change between the L1256F and wild-type ALK targeted by 6 clinical drugs (∆∆*G* = ∆*G*_L1256F_ − ∆*G*_WT_) based on 4 binding free energy calculation strategies.

Figure 8 shows the performance of the above 4 strategies, where different colors are used to represent the standard deviation of the predicted values. Compared with the result of previous section, all the methods show better performance, with the Pearson correlation of MM/PB(GB)SA close to 0.9, and the 5-replica TI strategy also achieves a Pearson correlation of ~0.7. Such a high accuracy may be due to the relatively few drugs used for the study, but it is no doubt that these methods are still worth adopting when calculating for the same mutation among different drugs. When considering the absolute error, TI performs much better, with the MUEs of TI_1-replica_ and TI_5-replica_ of 1.50 and 1.27 kcal/mol, respectively, compared with the MUEs of MM/GBSA_100ns&ε=1_ and MM/PBSA_100ns&ε=1_ of 2.42 and 1.96 kcal/mol, respectively. Nevertheless, the high correlation of the result based on MM/PB(GB)SA makes the discrimination (of drug resistance) between systems more clear. Therefore, we decided to use MM/PBSA for the following analysis in order to be consistent with the previous analyses.

**Fig. 9.**
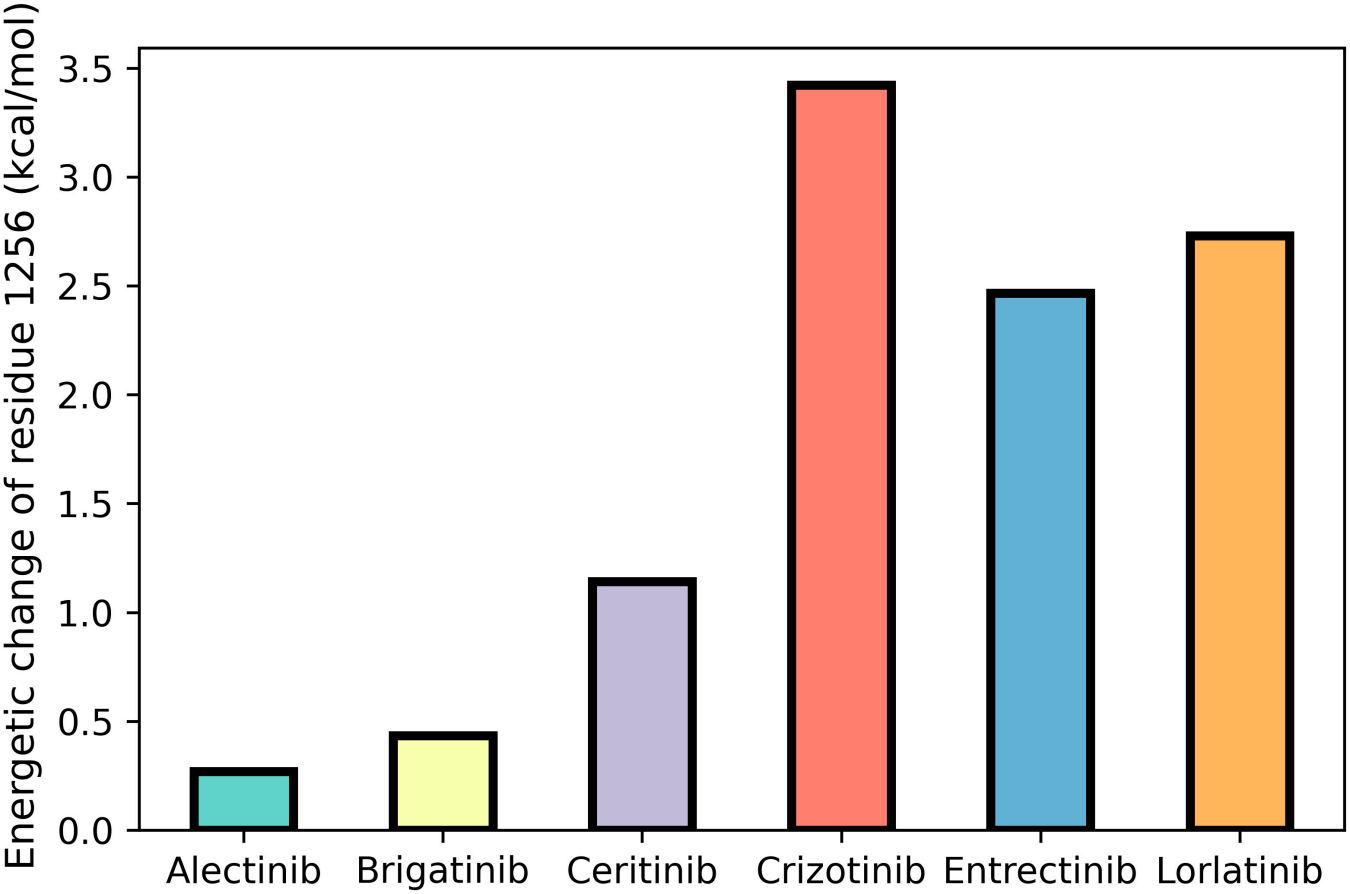
Energetic change of residue 1256 (L1256F) in the mutated and wild-type ALK, where a negative value represents the attenuated interaction between the drug and F1256 in the mutated ALK.

To find the key residues affecting the binding affinity of the drugs in the mutated system, we decomposed the total binding free energies and estimated the difference of residue–ligand interactions between the wild-type and the mutated systems (∆*G*_MT_ − ∆*G*_WT_). It is found that the mutation site itself (L1256F) brings the largest energetic difference to the drugs’ binding in 3 of 6 drugs, including crizotinib, entrectinib, and lorlatinib (Fig. 9). This means that phenylalanine (F1256) in the ALK mutant impacts the binding between the ligand and receptor directly, showing a shared resistance mechanism with the in-pocket mutation case (L1198F) analyzed above.

**Fig. 10.**
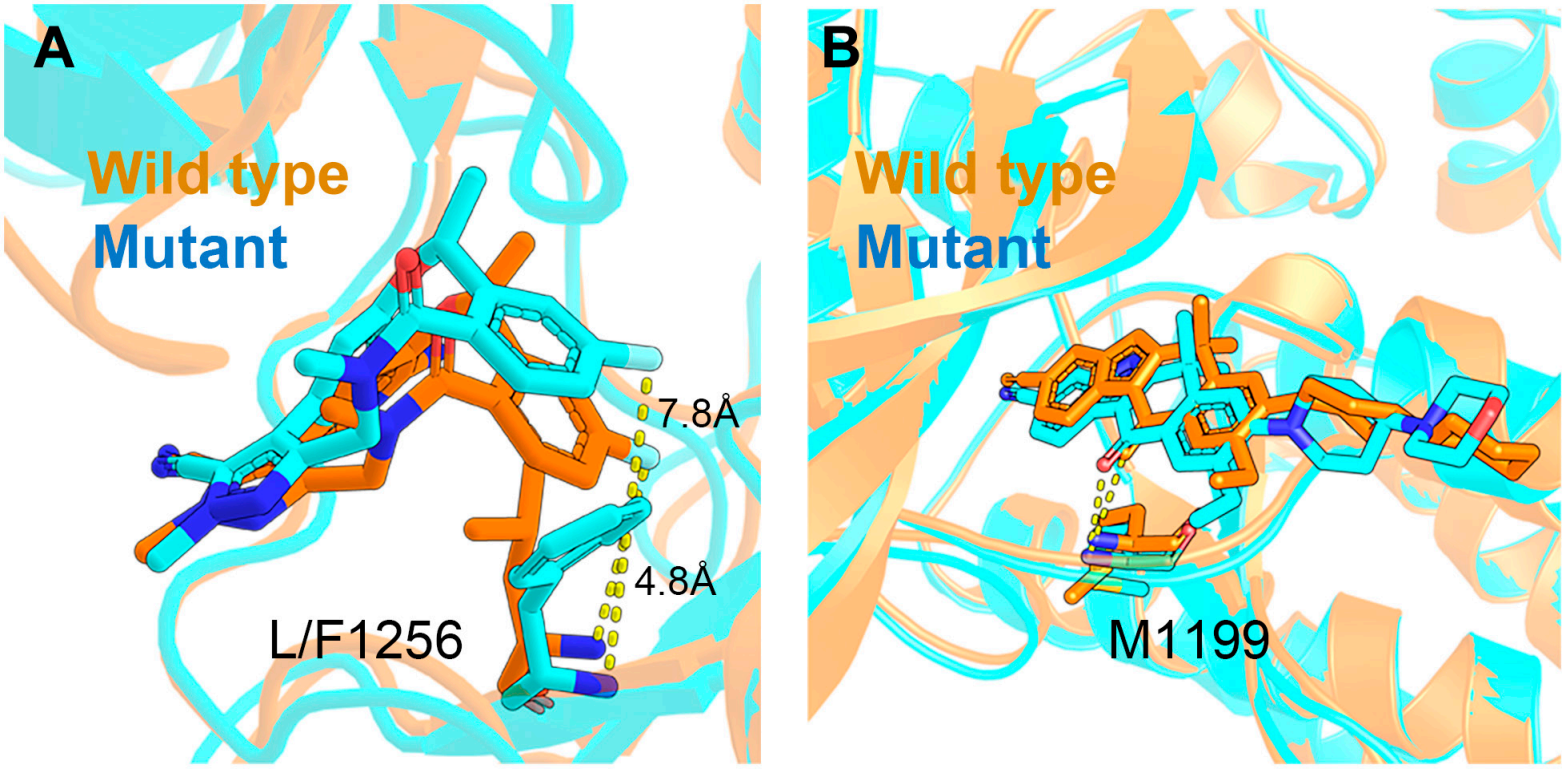
Average structures of lorlatinib (A) and alectinib (B) binding with the wild-type (orange) and L1256F (cyan) mutated ALK. The H-bond is shown in yellow dotted lines.

In the 6 systems, the system of ALK-lorlatinib can also be taken as an example for the detailed analysis. The average structures of the cMD simulations were compared for the wild-type and L1256F mutated systems. As shown in Fig. 10A, compared with the wild-type system (L1256, orange model), the side chain of phenylalanine (F1256, cyan model) in the mutant forces the benzene ring of lorlatinib farther from the mutation site, which makes the H-bond between the hydrogen on the backbone of F1256 and fluorine of lorlatinib broken (~8 Å; cyan stick model in Fig. 10A), resulting in the attenuated electrostatic interaction of ~1.0 kcal/mol (Table S6). Meanwhile, the position movement of the ligand also weakens its van der Waals interaction with the mutation site (F1256), leading to 1.36 kcal/mol energetic loss (Table S6).

Moreover, the surrounding residues near the mutation site may be affected by the mutation as well. Previous studies have reported that H-bonds between the ligand and the hinge region of ALK (purple region in Fig. 1) were important features for competitive TKIs’ binding [37]. Thus, the frequency of H-bonds in MD simulation was investigated to monitor the shift of the ligand in the mutated system. It shows that the H-bond between E1197 and lorlatinib exists in 1,294 frames of the MD trajectory (detected by cpptraj with default parameters) in the wild-type ALK-lorlatinib system, while the H-bond only exists in 927 frames in the L1256F mutant. Further investigation shows that the H-bond between E1197 and lorlatinib is broken in the latter half of the MD trajectory (~40 ns; Fig. S6A) with the distance between the 2 H-bond-forming atoms larger than 9 Å because of shift of the ligand in the mutated system (cyan stick model in Fig. S6B), which makes the electrostatic interaction loss of 4.65 kcal/mol to the residues 1197 to 1199 in L1256F mutant compared with lorlatinib in the wild-type ALK (Table S7). Besides, such a shift of the ligand also affects the van der Waals interaction of lorlatinib to the mutated ALK, making the total van der Waals and electrostatic interaction loss of 0.93 kcal/mol to the residues 1197 to 1199 in the mutated ALK, and eventually reflects on the total binding free energy difference.

However, it is shown that there is no significant energetic decrease in the L1256F mutant of the ALK-alectinib system. Further analysis shows that the drug in the trajectories of both systems go with a similar behavior (without large change) after the introduction of the mutation. Different from the lorlatinib’s macrocyclic structure, alectinib’s slender structure only results in the binding orientation change in the tail of the ligand in the mutated ALK (Fig. 10B), which does not affect the vital interactions to the protein. Compared with the wild-type protein, the H-bond between alectinib and M1199 does not change in the L1256F mutant, with the average H-bond distances of 2.16 and 2.28 Å, respectively, in the wild-type and mutated ALK (Fig. S7), making alectinib insensitive to the mutation as reported by the experimental data (Table S2).

## Conclusion

In this study, on the basis of investigating the performance of several widely used computational methods (MM/PB(GB)SA and TI), both the shared and specific drug resistance mechanisms were revealed to the ALK system.

In the one-drug-multiple-mutation cases (including 28 ALK-lorlatinib systems), 3 kinds of representative mutations (in-pocket, out-pocket, and double-mutation cases) were taken as examples to study the drug resistance mechanisms and furthermore deduce the shared drug resistance mechanisms, in which the in-pocket mutations usually lead to drug resistance by hindering the drug’s binding directly (e.g., the cases of L1198F and L1256F), whereas the out-pocket mutations may influence the drug’s binding by a more complicated way, such as with a long signaling transmission chain. Furthermore, the double-mutation (or multi-mutation) mutant may impair the drug’s binding with an aggregated (or cooperative) effect (e.g., the case of I1171S&G1269A), where it may, on the one hand, hinder the drug–target interaction directly and, on the other hand, influence the kinetic property of the protein structure as that found in the out-pocket mutations.

Moreover, the multiple-drug-one-mutation (L1256F) case was also investigated. The specific drug resistance mechanism was revealed by comparing the difference of binding sensitivity between lorlatinib and alectinib, where the different scaffolds of the 2 drugs result in the direct steric hindrance of the mutation to lorlatinib, but little influence to the binding of alectinib, showing a shared drug resistance mechanism of the in-pocket mutations and highlighting the structure specificity-guided combating-resistance drug design.

## Materials and Methods

### Structure preparation

The crystal structure of ALK accompanied with 6 drugs, including the first-generation ALK TKI crizotinib [Protein Databank (PDB) code 2XP2 [38]], the second-generation ALK TKIs alectinib (PDB code 3AOX [39]), ceritinib (PDB code 4MKC [40]), brigatinib (PDB code 6MX8 [41]), and entrectinib (PDB code 5FTO [42]), and the third-generation ALK TKI lorlatinib (PDB code 4CLI [43]), together with various mutants consists of the dataset of this study (Fig. 1), where the experimental activity data were derived from Mizuta’s work [36].

At the stage of system preparation, all the mutants were manually mutated by the Build and Edit Protein module in Discovery Studio/2019 [44], and the modified part of the protein was consequently relaxed by the geometry refinement and CHARMm force field optimization to eliminate structural bumps between the manually introduced mutations and the surroundings. The topology file was generated by the antechamber and tleap modules in AMBER/20 simulation package [45,46]. All the ligands were assigned with the AM1-BCC atomic charge due to its good performance in various applications [47–50]. The general AMBER force field (gaff/1.81) [51] and ff14SB force field [52] were employed to parameterize the ligand and protein, respectively. A 10Å-extended-cubic TIP3P [53] water box was added for each system to immerse the protein-ligand complex with counterions of Na^+^ or Cl^−^ used to neutralize the redundant charges of the systems.

### Molecular mechanics minimization

Molecular mechanics (MM) minimization was conducted before MD simulation to further relax the unfavorable contacts arising from imperfect crystallization or manually introduced mutations. At this stage, the real-space cutoff was set to 10 Å to handle the short-range van der Waals and electrostatic interactions, while the PME algorithm (particle mesh Ewald) [54] was implemented to calculate the long-range electrostatic interactions. MM minimization was performed for the systems before the long-time MD simulation as follows: (a) All the atoms except hydrogen were restrained; (b) heavy atoms in water (the oxygen atom) and counterions (Na^+^ or Cl^−^) were relaxed in addition; (c) the heavy atoms in side chain of residues and the ligand were set free additionally for optimization; (d) all the atoms were set free for full minimization. Five thousand steps of minimization, including 1,000 circles of steepest descent and 4,000 cycles of conjugate gradient minimizations, were conducted with the system constrained at 5 kcal/mol∙Å^2^ in the first 3 steps, while 10,000 steps (5,000 circles of steepest descent and conjugate gradient minimizations) were performed for the last step. All the MM minimizations were performed with the pmemd module in AMBER/20.

### cMD simulation

In the process of cMD simulation, a 3-step MD simulation is performed for each system as follows: (a) The system was gradually heated from 0 to 300 K within 50 ps in the NVT ensemble, in which all the heavy atoms in the backbone of protein were constrained with 2 kcal/mol∙Å^2^; (b) then, an equilibrium simulation was performed in the NPT ensemble (50 ps, *T* = 300 K, and *P* = 1 atm) with the same restraint as the previous step; (c) finally, 100-ns cMD simulation was carried out in the NPT ensemble (*T* = 300 K and *P* = 1 atm) with no restraint on the system. In all the MD simulations, the time step was set to 2 fs with the SHAKE algorithm [55] constraining the covalent bonds between the hydrogen atoms and the connected heavy atoms. The coordinates were collected with an interval of 5 ps, and a total of 2,000 frames were collected for each system. All the cMD simulations were performed with the pmemd.cuda module in AMBER/20.

### End-point binding free energy calculations with MM/GBSA and MM/PBSA

The MM/PBSA and MM/GBSA analyses were carried out based on the cMD trajectories. In the MM/PB(GB)SA approach, the binding free energy (∆*G*_bind_) upon ligand–receptor interaction (Eq. 1) can be decomposed into different energy terms according to the following formulas [56].

$$∆G_{\text{bind}}=G_{\mathrm{complex}}-\left(G_{\mathrm{receptor}}-G_{\mathrm{ligand}}\right)$$ (1)

$$∆G_{\mathrm{bind}}=∆H-T∆S=∆E_{\mathrm{MM}}+∆G_{\mathrm{sol}}-T∆S$$ (2)

$$\begin{array}{c} ∆E_{\mathrm{MM}}=∆E_{\mathrm{bond}}+∆E_{\mathrm{angle}}+\\ ∆E_{\mathrm{dihedral}}+∆E_{\mathrm{ele}}+∆E_{\mathrm{vdW}} \end{array}$$ (3)

$$∆G_{\mathrm{sol}}=∆G_{\mathrm{PB}/\mathrm{GB}}+∆G_{\mathrm{SA}}$$ (4)

$$∆G_{\mathrm{SA}}=\gamma *\mathrm{SASA}+b$$ (5)

where Δ*E*_MM_, Δ*G*_sol_, and −*T*Δ*S* represent the MM energy, the solvation free energy, and the entropy upon ligand binding, respectively (Eq. 2), in which Δ*E*_MM_ consists of 5 energy terms, namely, the bond (Δ*E*_bond_), angle (Δ*E*_angle_), dihedral (Δ*E*_dihedral_), electrostatic (Δ*E*_ele_), and van der Waals (Δ*E*_vdW_) energies (Eq. 3). Here, we applied the single MD trajectory protocol for the binding free energy calculation because it usually results in more stable result by canceling out Δ*E*_bond_, Δ*E*_angle_, and Δ*E*_dihedral_ in the following calculations [28]. Δ*G*_sol_ contains 2 parts, namely, the polar (Δ*G*_PB/GB_) and nonpolar (Δ*G*_SA_) contributions to the solvation free energy (Eq. 4), in which the polar part can be calculated by either the Poisson–Boltzmann (PB) equation or the Generalized Born (GB) model, while the nonpolar part is usually estimated from the solvent accessible surface area (SASA) using the LCPO algorithm [57]. Here, the PB model parameterized by Tan et al. (PB^pbsa^) [58] and the GB model proposed by Onufriev et al. (GB^OBC1^) [59] were employed for the polar solvation energy calculations (Δ*G*_PB/GB_). To investigate the dielectric effect to the performance of the resulted binding free energy since it significantly affects the electrostatic part (Δ*E*_ele_ and Δ*G*_PB/GB_) of the results [32,33,60], here 1, 2, and 4 were set for the solute (or interior) dielectric constant (ε_in_) to give a comparison, while the solvent (or outer) dielectric constant was set to 80. The nonpolar solvation energy (Δ*G*_SA_) was calculated with γ and b set to 0.0072 and 0, respectively (Eq. 5). Normal mode analysis (NMA) was conducted to estimate the conformational entropy change (−*T*∆*S*) of the system (termed as NME). To save the computational resources, the structure–truncation strategy was used to speed up the NME calculation [61]. Here, a 9-Å cutoff was set to truncate the protein around the ligand as its reasonable performance [32]. To keep complete of the associated residues, the whole residue will be incorporated into the truncated structure if any heavy atoms of the residue drop into the cutoff sphere. The terminal of discontinuous residues was treated with charged groups (COO^−^ and NH_3_^+^) because of its better performance [32]. Fifty frames extracted with an equal interval from the 100-ns cMD trajectory (namely, 2 ns/frame) were submitted for NME calculation, where the maximum optimizing steps and convergence condition were set to 10,000 and 1 × 10^−4^, respectively. All the MM/PB(GB)SA/NME calculations were conducted with MMPBSA.py [62] in AMBER/20.

### Umbrella sampling

Theoretically, it is possible to explore the binding/dissociation details of drug–target interaction with cMD simulation. However, it usually needs huge amount of sampling to observe the transformation between 2 thermodynamic states divided by high energetic barriers. Fortunately, importance sampling methods [63], such as US [64], metadynamics (MetaD) [65], and adaptive biasing force (ABF) [66], that work by focusing on the specific reaction coordinate (RC; ξ) can greatly accelerate the sampling process. Here, we employed US to seek possible transfer chain of the mutation effect of the ALK-lorlatinib system.

In the spirit of US algorithm, it works by adding biasing potential to the RC to drive the system from one thermodynamic state to another. In practice, to facilitate convergence of the system, a series of simulation windows are usually divided along the RC with a harmonic biasing potential added to each window according to (Eq. 6).

$$w_{i}\left(\xi \right)=\frac{K}{2}{\left(\xi -\xi_{i}^{\text{ref}}\right)}^{2}$$ (6)

where *ω*_*i*_(*ξ*) represents the biasing potential with an elastic constant of 𝐾 added between the current position (*ξ*) and the reference position (*ξ*^*ref*^) in window *i* of the RC.

The US simulation was initiated from the conformation of the minimized structure of the investigated systems. Here, the RC was defined by the distance between CB of E1167 and C8 of the ligand in the starting conformation. The RC was designed to dissociate the ligand from 12.5 Å (in its bound state) to 32.5 Å (in bulk solvent) during 41 windows, with each window covering 0.5 Å of the RC. A harmonic elastic constant of 5 kcal/mol·A^2^ was set in the middle of each window to help the ligand overcome the energetic barriers located in the RC. For each round of US simulation, 1-ns US sampling was conducted at 300 K in the NTP ensemble, and a total of 18 rounds of US simulation (738 ns) was performed for each system to guarantee convergence of the system (namely, 1 ns × 41 windows × 18 rounds; Fig. S1). The weighted histogram analysis method (WHAM) [67,68] was used to calculate the PMF of the ligand dissociating along the RX. All the US simulations were performed with the pmemd.cuda module of AMBER/20.

### Thermodynamic integration (TI)

Compared with the end-point method represented by MM/PB(GB)SA approach, alchemical method of thermodynamic integration (TI) and free energy perturbation (FEP) computes binding free energy with more rigorous theory. It is generally believed that the alchemical method can generate more accurate result compared with the end-point binding free energy calculation methods. Therefore, here, TI was additionally employed to verify the calculation accuracy of the ALK systems.

In the algorithm of TI, the free energy difference between 2 states of a system (∆*G* = *G*_1_ − *G*_0_) can be expressed according to Eq. 7,

$$∆G=\int_{0}^{1}\langle \frac{\partial U\left(\lambda \right)}{\partial \lambda }\rangle$$ (7)

$$U\left(\lambda \right)=U_{0}+\lambda \Delta U$$ (8)

where 0 represents the initial state, while 1 represents the final state. *U*(λ) is the potential energy of the intermediate state λ (0 < λ < 1), with ∆*U* representing the potential energy difference between 2 adjacent states (namely, ∆*U* = *U*_1_ − *U*_0_), which can be integrated from the initial state (λ = 0) to the final state (λ = 1) to derive the total free energy change in the process. In practice, ∆*G* can be calculated by multiple methods, such as Trapezoidal Rule (TRA) [69], Gauss-Legendre Quadrature (GAU) [70], Clenshaw-Curtis Integration (CC) [71], Bennett Acceptance Ratio (BAR) [72], and Multistate Bennett Acceptance Ratio (MBAR) [73]. Here, we used the TRA method to calculate ∆*G*. The TI simulation was started with the 100-ns equilibrated MD structures (*T* = 300 K and *P* = 1 atm). Softcore was used to avoid abnormal value in the computation of van der Waals (Lennard–Jones) and electrostatic (Coulombic) interactions [74,75]. The time step was set to 1 fs without constraining any covalent bonds involving hydrogen atoms. For each system, 14 λ simulations (λ = 0, 0.0001, 0.001, 0.01, 0.1, 0.2, 0.3, 0.4, 0.5, 0.6, 0.7, 0.8, 0.9, and 1.0) were performed sequentially with the van der Waals and electrostatic interactions switching on/off simultaneously for both the holo-state and apo-state computations. Each λ was run for 1 ns, and 5 replicas were conducted for each system to gain an average binding free energy. All the TI computations were performed with the pmemd.cuda module in AMBER/20.

### Contact network analysis

In CNA, the Cα atoms were defined as nodes, in which the communication of residues within 4.5 Å of a given node was considered as edges, with the edge weight (*d_ij_*) between 2 nodes calculated according to Eq. 9,

$$d_{\mathrm{ij}}=-\log\left(C_{\mathrm{ij}}\right)$$ (9)

$$C_{\mathrm{ij}}=\frac{<\;∆r_{i}\times \;∆r_{j}\;>}{\sqrt{\left(<\;∆{r_{i}}^{2}\;><\;∆{r_{j}}^{2}\;>\right)}}$$ (10)

where *i* and *j* represent 2 communicating nodes, and *C_ij_* represents pairwise correlation defining the probability of information transferring across a given edge. ∆*r_i_* denotes the fluctuation of atom *i* (Cα of a residue) to the original position in the whole trajectory (Eq. 10). CARMA [76] was used to calculate the correlation of the nodes, and the Girvan Newman algorithm was used to obtain the network map [77]. All the CNA was conducted using the network view plugin in VMD [78,79].

## Data Availability

AMBER/20 was used for all the computations, including MD simulation, end-point, alchemical, and US binding free energy calculations. The structures were drawn with PYMOL/2.4.1 education edition, and the scatter diagrams were plotted with R/3.6.1. The data are available from the authors upon a reasonable request.
