# Supplementary material for: Deciphering the Shared and Specific Drug Resistance Mechanisms of Anaplastic Lymphoma Kinase via Binding Free Energy Computation
Source: Research (Wash D C). 2023 Jun 19;6:0170. doi: 10.34133/research.0170 (PMC10278961; doi:10.34133/research.0170)
Supplement: Supplementary 1 — Tables S1 to S7 Figs. S1 to S7 [file research.0170.f1.pdf]

Supporting information

Deciphering the Shared and Specific Drug Resistance Mechanisms of Anaplastic Lymphoma Kinase via Binding Free Energy Computation

Yang Yu[1,#], Zhe Wang[2,#], Lingling Wang[1], Qinghua Wang[1], Rongfan Tang[1], Sutong Xiang[1], Qirui Deng[1], Tingjun Hou[2,*] and Huiyong Sun[1,*]

[1]Department of Medicinal Chemistry, China Pharmaceutical University, Nanjing 210009, Jiangsu, P. R. China.

[2]Innovation Institute for Artificial Intelligence in Medicine of Zhejiang University, College of Pharmaceutical Sciences, Zhejiang University, Hangzhou 310058, Zhejiang, P. R. China.

[#]These authors contributed equally to this work.

Table S1. Detailed information of the ALK mutants ($\Delta\Delta G_{exp}$=ΔG_{MT}-ΔG_{WT}, kcal/mol).

mutant	$\Delta\Delta G_{exp}$	mutant	$\Delta\Delta G_{exp}$	mutant	$\Delta\Delta G_{exp}$	mutant	$\Delta\Delta G_{exp}$
C1156Y	0.77	G1202R	2.69	I1171N-G1269A	3.71	I1171T	1.70
D1203N	1.46	G1202R-L1196M	4.02	I1171N-L1196M	3.38	L1196M	2.44
D1203N-F1245V	1.91	G1202R-L1198F	3.31	I1171N-L1198F	3.47	L1196Q	1.97
D1203N-L1196M	3.20	G1269A	1.50	I1171N-L1198H	3.38	L1198F	1.46
F1174I	1.26	I1171N	2.54	I1171N-L1256F	4.95	L1256F	5.28
F1174V	0.97	I1171N-F1174I	3.35	I1171S	2.05	T1151K	0.82
F1245V	0.46	I1171N-F1174L	2.03	I1171S-G1269A	3.90	V1180L	0.39

Table S2. Experimental binding free energy difference between the wild-type and L1256F mutated ALK in the six drugs (kcal/mol).

**Drug**	$\Delta\Delta G_{exp}$
Alectinib	-0.61
Brigatinib	1.03
Ceritinib	2.20
Crizotinib	1.78
Entrectinib	2.88
Lorlatinib	5.28

Table S3. Energetic contribution of residue 1198 in the wild-type and L1198F mutated ALK based on MM/PBSA result (kcal/mol).

**Lorlatinib**	ΔE_{vdW}	ΔE_{ele}	ΔG_{PB}	ΔG_{SA}	ΔG_{total}
L1198-WT	-2.83	-2.29	1.41	-0.12	-3.83
F1198-MT	-2.65	-0.93	1.30	-0.12	-2.40
$\Delta\Delta G_{MT\text{-}WT}$	0.19	1.35	-0.16	0.00	1.43

Table S4. MM/PBSA result of the wild-type and 1171S mutated ALK binding with lorlatinib (kcal/mol).

**Replica**	**WT**	**I1171S**	$\Delta\Delta G$
1	-38.54	-36.68	1.86
2	-38.60	-37.75	0.85
3	-38.06	-36.25	1.81

Table S5. Energetic change of vital residues in the wild-type and 1171S mutated ALK to lorlatinib based on MM/PBSA result (kcal/mol).

**Residue**	**WT**	**MT**	$\Delta\Delta G_{MT\text{-}WT}$
L1122	-1.51	-1.94	-0.44
E1132	0.27	0.83	0.55
A1148	-0.26	-0.53	-0.27
K1150	-0.10	1.01	1.11
E1197	0.21	0.65	0.44
L1198	-0.21	-0.96	-0.75

M1199	-0.22	-0.47	-0.25
A1200	-0.37	-0.67	-0.31
D1203	1.94	1.17	-0.77
S1206	-1.02	-0.19	0.83
R1209	-1.25	-0.11	1.14
R1212	-0.60	0.09	0.69
R1214	-0.84	-0.01	0.83
N1254	0.10	-0.16	-0.25
L1256	-1.01	-1.28	-0.28
Y1327	-0.09	-0.33	-0.24
E1400	0.82	0.13	-0.69

Table S6. Energetic contribution of residue 1256 in the wild-type and mutated ALK-lorlatinib systems (kcal/mol).

	ΔE_{vdW}	ΔE_{ele}	ΔG_{PB}	ΔG_{SA}	ΔG_{total}
L1256-WT	-5.52	-0.33	0.36	-0.64	-6.13
F1256-MT	-4.16	0.66	0.82	-0.72	-3.40
$\Delta\Delta G_{MT-WT}$	1.36	0.99	0.46	-0.09	2.72

Table S7. Energetic contribution of Residues 1197-1199 in the wild-type and L1256F mutated ALKs (kcal/mol).

	ΔE_{vdW}	ΔE_{ele}	ΔG_{PB}	ΔG_{SA}	ΔG_{total}
E1197-WT	-0.50	-3.53	0.70	-0.02	-3.35
L1198-WT	-2.83	-2.29	1.41	-0.12	-3.82
M1199-WT	-3.10	-4.43	4.29	-0.14	-3.37
E1197-MT	-0.27	-1.46	0.31	-0.02	-1.44
L1198-MT	-2.70	-2.21	1.71	-0.12	-3.33
M1199-MT	-2.99	-4.25	4.97	-0.30	-2.57
$\Delta\Delta G_{1197-1199}$	0.93	4.65	0.58	-0.34	3.20

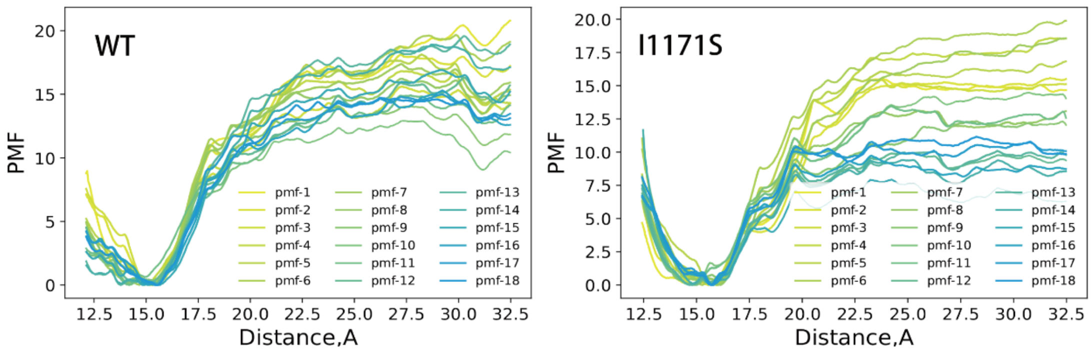

Figure S1. Convergence of the PMFs in US simulation.

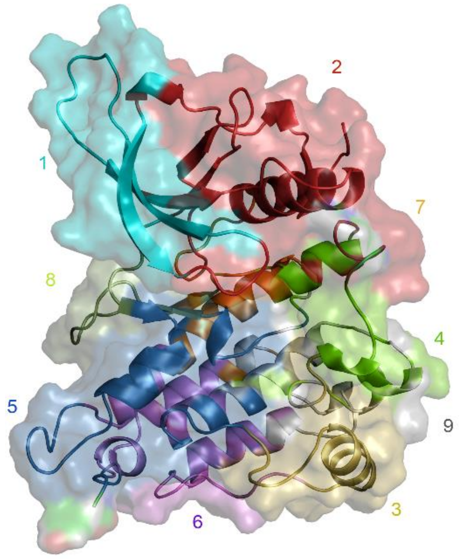

Figure S2. Community distribution of the wild-type system.

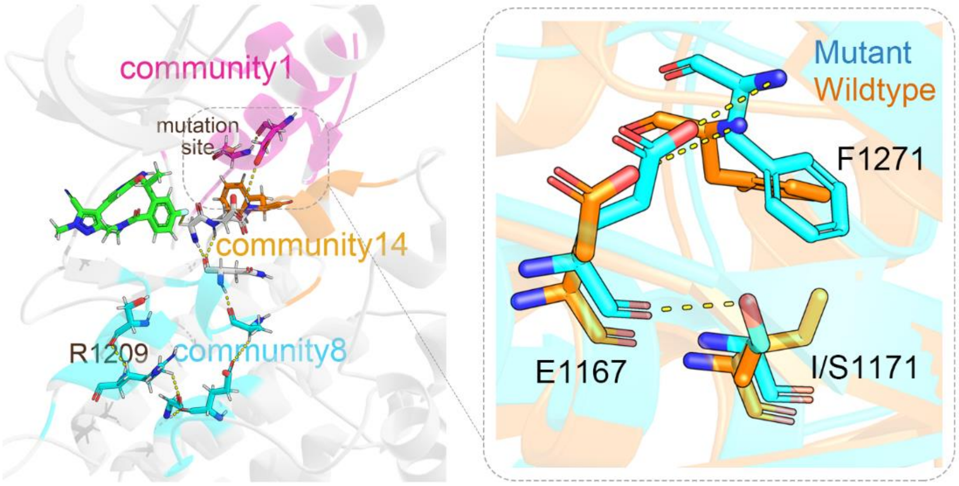

Figure S3. H-bond between S1171 and E1167 in the I1171S mutated ALK.

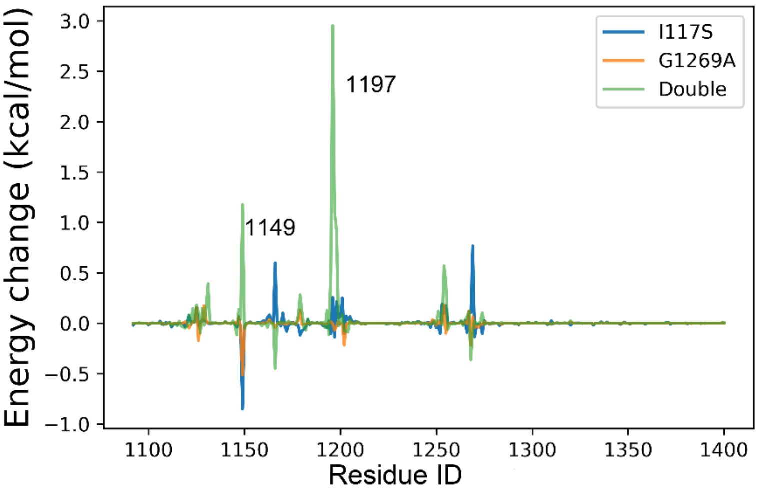

Figure S4. Difference of the energetic decomposition spectra between the wild-type and mutated ALK-lorlatinib systems (ΔG_{MT}-ΔG_{WT}).

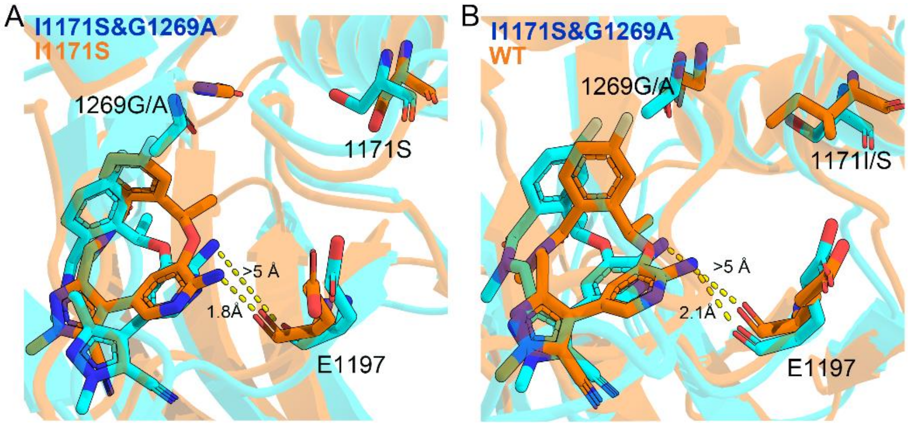

Figure S5. The averaged binding mode of lorlatinib in the wild-type (orange, panel B), I1171S (orange, panel A) and G1269A&I1171S (cyan, panel A and B) systems of ALK, where the H-bond is colored in yellow dashed line.

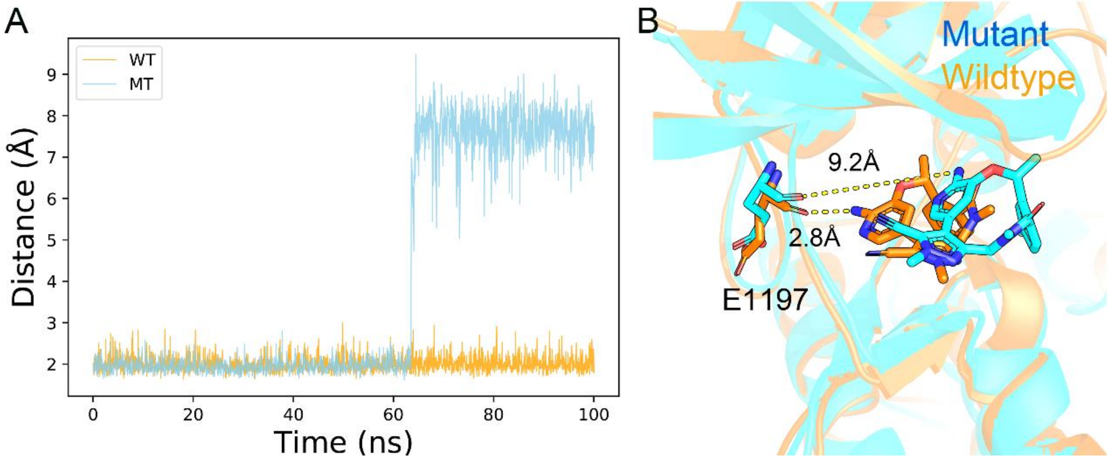

Figure S6. (A) Evolution of H-bond between lorlatinib and E1197 during the 100 ns MD simulation in the wild-type (orange) and L1256F mutated (cyan) ALK. (B) The average conformations of the ligand in the last 40 ns cMD trajectories are illustrated in the two systems, where a clear shift is shown of the drug in the L1256F mutated ALK (cyan stick model) with the H-bond broken (>9 Å).

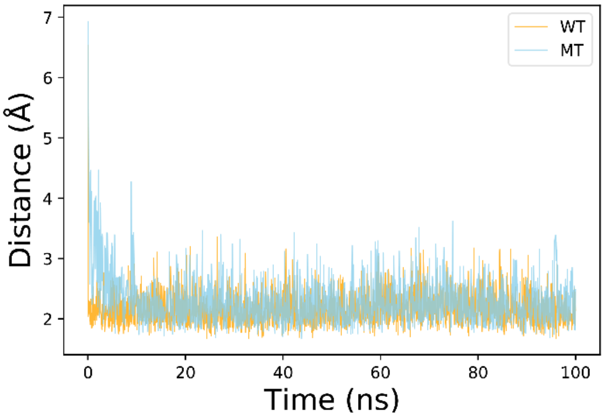

Figure S7. Evolution of H-bond between alectinib and M1199 during the 100 ns MD simulation in the wild-type (orange) and L1256F mutated (blue) ALK.
